# Internal Fixation of Transverse Patella Fractures Using Cannulated Cancellous Screws with Anterior Tension Band Wiring

**DOI:** 10.5704/MOJ.1607.005

**Published:** 2016-07

**Authors:** I Khan, MY Dar, S Rashid, MF Butt

**Affiliations:** Govt Medical College Jammu, Jammu and Kashmir, India

**Keywords:** Patella, transverse fracture, open reduction, cannulated screw, anterior tension band

## Abstract

**Aims**: To evaluate the effectiveness and safety of anterior tension band wiring technique using two cannulated cancellous screws in patients with transverse (AO34-C1) or transverse with mildly comminuted (AO34-C2) patellar fractures.

**Materials and Methods:** This is a prospective study of 25 patients with transverse fracture or transverse fracture with mildly comminuted patella fractures. All the patients were treated with open reduction and internal fixation using two parallel cannulated screws and 18G stainless steel wire as per the tension band principle.

**Results**: There were eighteen males (72%) and seven females (28%). The age group ranged from 24 to 58 years, with mean age of 38 years. The most common mode of injury was fall (72%) followed by road traffic accident (20%) and violent quadriceps contraction (8%). Transverse fracture was present in 60% and transverse fracture with mild comminution in 40% of patients. Mean time to achieve union was 10.7 weeks (range 8-12 weeks). Mean ROM at three months was 113.8 degree (90-130) and at final follow up this improved to 125.4 degrees (range 100-140). There was one case of knee stiffness and no case of implant failure was observed. Patients were evaluated using Bostman scoring, the mean score at three months being 26.04 which improved to 27.36 at the end of final follow up at one year.

**Conclusion**: Cannulated cancellous screws with anterior tension band wiring is a safe, reliable and reproducible method in management of transverse patellar fractures, with less chances of implant failure and soft tissue irritation.

## Introduction

The patella plays an important role in knee joint motion. Patellar fractures are mostly seen in the age group of 20-50 years, comprising about 1% of all skeletal injuries^1, 2^. To preserve the range of knee motion adequate management followed by aggressive post-operative rehabilitation is mandatory. Patellar fractures may be displaced or undisplaced. Undisplaced fractures are usually managed by immobilizing the limb in a cylindrical cast and allowing mobilization as tolerated by the patient provided the extensor mechanism is intact. However, the displaced patellar fractures are usually managed surgically to minimize the risk of developing post-traumatic arthritis^3^, necessitating open reduction. The surgical management of patellar fractures has evolved over the years from simple cerclage wiring to tension band wiring (TBW)^4^ which was further modified to increase its strength. Cancellous screws have also been used in the management of transverse patellar fractures, with high failure rates compared to modified tension band wiring. The addition of anterior tension band wiring with the cannulated cancellous screws has added strength to the construct. The surgical management of patellar fractures is constantly evolving, with the latest additions being cable pin system^5^, mini-screw fragment fixation system^6^ and fixed angle plate fixation^7^ for various patellar fracture configurations. The evolution in patellar fracture management has been necessitated by the need for stable fixation to allow early mobilization and aggressive rehabilitation to preserve the range of knee motion in the young physically active age group.

The revolution in the management of patellar fractures was brought on with the introduction of tension band wiring^4^ in 1950s, which was further modified by adding Kirschner wires (K-wires) to the construct to increase its strength, allowing early mobilization and rehabilitation. The K-wires may protrude at times leading to soft tissue irritation, hindering the range of knee motion. Since the knee is immobilized in extension in the early post-operative period and early mobilization, the quadriceps pull is straight and thus may exert traction on the K-wires leading to loss of reduction. The sharp ends of the K-wires distally may hinder kneeling, thus hampering certain religious activities which require kneeling during praying in the Indian population. To overcome these shortcomings a new technique has evolved replacing the K-wires with cannulated cancellous screws. The screws act as lag screws compressing the fracture site. Mechanically, the addition of the screws to the tension band techniques reduces fracture separation by providing compression throughout the range of motion and by resisting the tensile loading during terminal extension^8^. The construct has been proved to be mechanically stronger than modified tension band in various biomechanical studies pioneered by Burvant *et al*^8^ and followed by Carpenter *et al*^9^ and Cekin *et al*^10^. Various clinical studies have also confirmed these findings.

## Materials and Methods

This is a prospective clinical study of 25 patients enrolled after obtaining institutional ethical clearance. All the selected patients were informed about this new surgical technique and formal consent was obtained. Of the 25 patients enrolled, eighteen were males (72%) and seven females (28%), the male majority consistent with other studies. The average age in our study was 38 years (range 24-58 years) The left knee (68%) was involved more frequently than the right (32%). Most common mode of injury was fall (72%), followed by RTA (20%) and violent quadriceps contraction (8%). Transverse fractures without comminution (AO 34-C1) were present in 15 patients (60%) and transverse fractures with mild comminution (AO 34-C2) were present in ten patients (40%). Three-part fractures and comminuted fractures are not amenable to screw fixation and were excluded from the study.

**Fig. 1:**
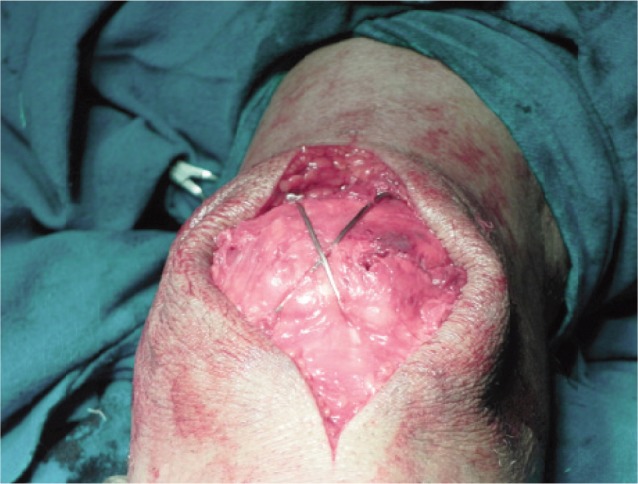
Intraoperative picture of the final construct.

Under tourniquet control a longitudinal midline incision was used to expose the fracture site. The retinacular tears were identified and the knee joint was inspected for loose fragments and cartilage damage (osteochondral fractures). The joint was irrigated and debrided of irreparable bone fragments. The fracture fragments were anatomically reduced and held using reduction clamp or towel clips. Articular congruity was assessed by digital palpation through the retinacular tear and confirmed on fluoroscopy. Two parallel guide wires were passed and their position checked under image intensification. After confirming the screw size, the screws were passed over the guide wires in antegrade or retrograde direction after drilling the fracture fragments. The guide wires were removed and an 18G stainless steel wire was passed through the cannulated screws and crossed over the anterior aspect of the patella. The wire ends were tightened with the knee in full extension. The articular surface was evaluated by palpating the articular surface through the retinacular defect and by fluoroscopy. To compress the fracture site the wires were sequentially tightened both medially and laterally. Final stability of the construct was tested by taking the knee through the range of motion (Fig. 1). Finally the soft tissues were repaired including the synovium, capsule and extensor mechanism and the wound closed and the knee immobilized in a hinged knee brace in extension.

Early physiotherapy comprising of passive ROM in a hinged knee brace set at 0-30 degree were started on the second post-operative day. Static quadriceps and hamstring setting were taught and weight bearing using crutches allowed as tolerated. Gradually the ROM was increased up to 90 degrees by the second week and increased as tolerated by the patient.

**Fig. 2:**
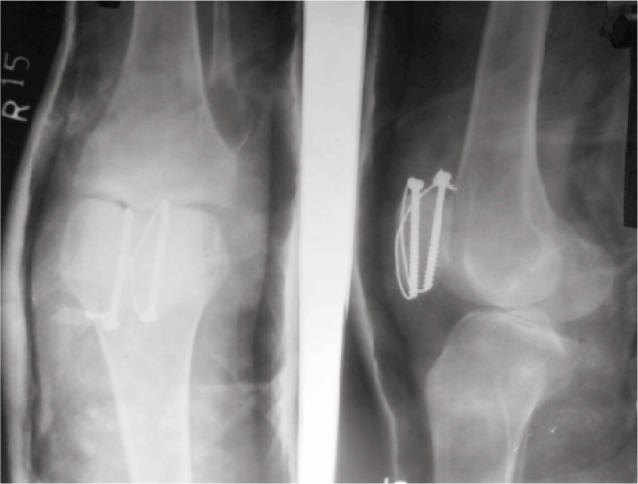
Postoperative **(A)** anteroposterior and **(B)** lateral radiographs. Demonstrating fracture fixation with cannulated cancellous screws and anterior tension band.

The patients were followed up at two and six weeks, three months and one year. Both clinical and radiological assessments were done for fracture healing and functional recovery (Figure 2A, 2B). Loss of fixation was defined as a gap at fracture site more than 3mm or articular step more than 2mm. Radiological union was established when the bony trabeculae crossed the fracture line. Patients were evaluated using the Bostman scoring.

## Results

**Fig. 3:**
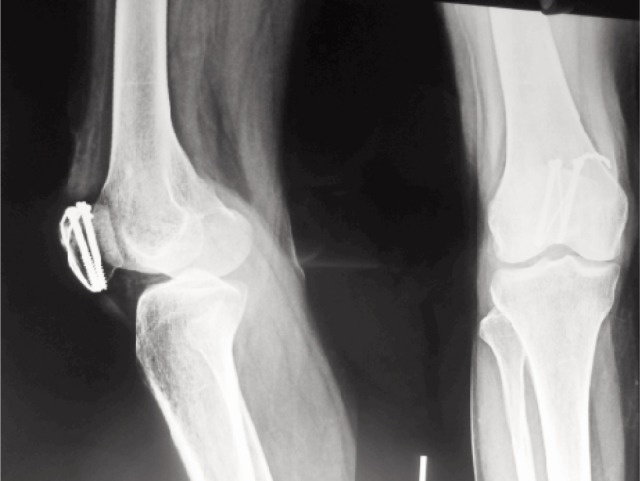
Radiographs demonstrating union at the fracture site (in this patients at 10 weeks) **(A)** Lateral **(B)** Anteroposterior radiograph.

**Fig. 4:**
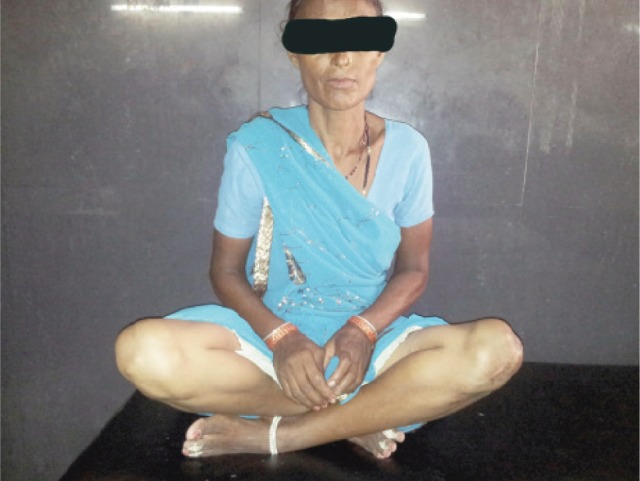
Demonstrating full range of motion at one year follow up.

Mean time to achieve radiological union was 10.7 weeks after the surgery (range 8-12 weeks) (Fig. 3A, 3B). Mean ROM at three month follow up was 113.8 (range 90-130-degrees) and at the final follow up at one year was 125.4 degrees (range 100-140 degrees) (Figure 4). The mean Bostman score at three months was 26.04 which improved to 27.36 at the end of final follow up at one year (Table I).

**Table I:** Depicting patient characteristics, fracture configuration, ROM at 3 months and one year follow up and *Bosmann* sco ring at 3 months and one year

										BOSTMAN SCORING	
S. NO.	AGE	SEX	SIDE	MODE OF INJURY	PATTERN OF RO FRACTURE	M AT THREE MONTHS	FINAL ROM	RADIOLO GICAL UNION	COMPLICATIONS	THREE MONTHS	ONE YEAR
1	48	M	L	Simple fall	C1	115	130	12	Superficial wound infection	27	30
2	42	M	L	Simple fall	C2	110	125	10		27	30
3	35	M	R	R.T.A	C1	130	140	8		30	30
4	52	M	L	Simple fall	C1	100	110	12		27	30
5	39	F	R	Simple fall	C2	90	100	10	Knee stiffness	24	24
6	49	M	L	Simple fall	C2	105	115	12		27	27
7	45	M	L	Simple fall	C1	125	135	11		27	27
8	30	M	L	R.T.A	C1	110	120	12		23	26
9	36	F	L	Simple fall	C2	125	140	12		29	29
10	55	M	R	Simple fall	C1	115	125	10		21	24
11	27	M	L	R.T.A	C2	105	125	8		21	24
12	38	F	L	Simple fall	C1	120	135	10		29	29
13	45	M	R	Simple fall	C2	105	115	12		23	23
14	28	M	L	Simple fall	C1	120	140	9		29	29
15	32	F	L	R.T.A	C2	120	135	10		30	30
16	46	M	R	Simple fall	C1	130	140	12		30	30
17	40	F	L	Simple fall	C1	120	135	10		28	28
18	58	M	R	Forced flexion	C1	105	120	12		20	23
19	24	M	L	R.T.A	C2	120	140	9		30	30
20	50	F	L	Simple fall	C1	100	115	12		27	27
21	47	M	L	Simple fall	C1	110	125	10		20	23
22	29	M	R	Forced flexion	C1	125	140	10		30	30
23	48	M	L	Simple fall	C2	120	130	12		24	24
24	51	M	R	Simple fall	C1	110	125	12		24	27
25	39	F	L	Simple fall	C2	110	125	12		27	30

R.T.A = ROAD TRAFFIC ACCIDENT ROM = RANGE OF MOTION

## Complications

Only two complications were observed. One case of superficial wound infection subsided with meticulous wound care and antibiotic administration. The second case had knee stiffness. This patient had an attack of acute pancreatitis for which she was hospitalized for a prolonged duration. This patient also gained useful ROM over a period of six months. No case of loss of reduction, implant migration or soft tissue irritation was observed.

## Discussion

The application of cannulated cancellous screws with anterior tension band wiring is a relatively new technique in the management of transverse patella fractures. The first biomechanical study was done by Burvant *et al*^8^ who compared five methods of fixation of patella fractures including modified TBW, anterior tension band with supplemental cerclage wiring (Pyrford technique), anterior tension band with cannulated cancellous screws, Pyrford technique with cancellous screws and cancellous screws alone. The technique of tension band with screws performed significantly better than the modified tension band. The second biomechanical study done by Carpenter *et al*^9^compared the mechanical effectiveness of three different techniques for stabilization of transverse fractures of the patella (a) modified tension band (AO technique); (b) two parallel 4.5-millimeter interfragmentary lag screws; or (c) a new technique using four-millimeter cannulated lag screws with a tension band wired through the screws. Fractures stabilized with a modified tension band were found to displace significantly more than those fixed with screws alone or screws plus a tension band in simulated knee extensions (p < 0.05). The fractures fixed with the cannulated screws plus the tension band failed at higher loads (mean = 732 newtons) than those stabilized with screws alone (mean = 554 newtons, p = 0.06) or those with a modified tension band (mean = 395 newtons, p < 0.05). The study concluded that combining interfragmentary screw fixation with the tension band principle appeared to provide improved stability over the modified tension band or screws alone for transverse patellar fractures. Cramer and Moed^12^ and Cekin *et al*^10^ in their biomechanical studies also substantiated the findings of the above studies.

The first clinical study done by Berg^13^ included 10 patients, and followed by Chang^14^ which also included 10 patients. In this study no patient had loss of fracture reduction, implant migration, material failure, or soft tissue irritation. The average Bostman score was 28.7 out of 30 (range, 27-30). The study concludes that anterior tension band wiring through cannulated screws for displaced inferior pole patella fractures is a safe, simple, and reliable alternative treatment with minimal soft tissue irritation. The clinical study by Malik and Halwai^15^ with a larger sample size, also confirmed the findings of the above mentioned studies. Chiang^16^ performed this technique arthroscopically and concluded that this was a safe and reproducible method for transverse patellar fractures. Jin^17^ performed this technique percutaneously and concluded that this technique provided stable fixation, allowed early motion exercise by minimizing injury to extensor mechanism and reduced cosmetic scar problems. Qi^18^ in his study using bioabsorbable cannulated lag screws and braided polyester suture tension bands concluded that this new double fixation technique resulted in satisfactory outcomes for patellar fractures without any obvious complications. The first comparative study was done by Tian^19^, though being a retrospective study, and it concluded that the titanium cable-cannulated screw tension band group showed improved fracture reduction, reduced healing time, and better Iowa score, compared with the modified tension band group. In the modified tension band group, eight patients experienced wire migration, three of these requiring a second operation. There were no complications in the titanium cable-cannulated screw tension band group. Hoshino^20^ compared the incidence of complications after tension-band fixation of the patella with Kirschner wires as compared with cannulated screws and observed Symptomatic implants, as the most common complication, were twice as frequent in patients treated with Kirschner wires. LeBrun^21^ evaluated the midterm functional outcomes of patients with isolated operatively treated patella fractures. The study concluded that at a mean of 6.5 years after operative treatment for patella fractures, significant symptomatic complaints and functional deficits persisted.

We here present our experiences with the open technique which is relevant in the Indian setup. In our study the males outnumbered the females which is consistent with the literature. The most common mode of injury was simple fall and the more common side involved was the left. Majority of fractures (60%) were simple fractures (AO 34-C1). The average time to achieve radiological union was 10.7 weeks. The average ROM at three months was 113.8 and the average Bostman score being 26.04. At the final follow up at one year average ROM improved to 125.4 and the average Bostman score being 27.36. No cases of implant failure, implant migration or soft tissue irritation were observed. One patient developed knee stiffness which improved over six months with useful range of motion regained.

This relatively new technique is a good alternative to modified tension band wiring. The construct being biomechanically stronger allows early regaining of full or useful range of motion, with less chances of implant failure and soft tissue irritation, thus minimizing the need for a second surgery. The drawback with our study is that it is not a comparative study and short follow up. We need prospective comparative studies with longer follow up to establish these advantages.

## Conclusion

Cannulated cancellous screws with anterior tension band wiring is a safe, reliable and reproducible method in the management of transverse patellar fractures, with less chances of implant failure and soft tissue irritation. This procedure is thus a good alternative to modified tension band wiring.

There is no conflict of interest in this study.
